# Bis(*N*′-benzoyl­pyridine-4-carbohydrazide)(1,10-phenanthroline)copper(II) dinitrate

**DOI:** 10.1107/S1600536810038985

**Published:** 2010-10-02

**Authors:** Xiu-Qing Zhang, Chun-Ying Li, He-Dong Bian, Qing Yu, Hong Liang

**Affiliations:** a College of Chemistry and Bioengineering, Guilin University of Technology, Guilin 541004, People’s Republic of China; b Key Laboratory for the Chemistry and Molecular Engineering of Medicinal Resources (Ministry of Education of China), School of Chemistry & Chemical Engineering of Guangxi Normal University, Guilin 541004, People’s Republic of China

## Abstract

In the title complex, [Cu(C_13_H_11_N_3_O_2_)_2_(C_12_H_8_N_2_)](NO_3_)_2_, the Cu^II ^atom (site symmetry 2) is coordinated by four N atoms from one 1,10-phenanthroline and two hydrazine ligands, respectively. The hydrazine ligands coordinate to the Cu^II^atom by a pyridine N atom. These four atoms form a slightly distorted square-planar N_4_ donor set. In the packing, two additional Cu⋯O inter­actions occur [Cu⋯O = 2.462 (2) Å], resulting in a typical Jahn–Teller-distorted octahedral environment around the Cu atom. N—H⋯O hydrogen bonds result in a three-dimensional network. The O atoms of the anion are disordered over two positions in a 0.68 (2):0.32 (2) ratio.

## Related literature

For general background to Schiff base complexes, see: Hursthouse *et al.* (1979 ▶); Gallego *et al.* (1979 ▶); Haran *et al.* (1980 ▶); Bian *et al.* (2005 ▶); Yu *et al.* (2006 ▶).
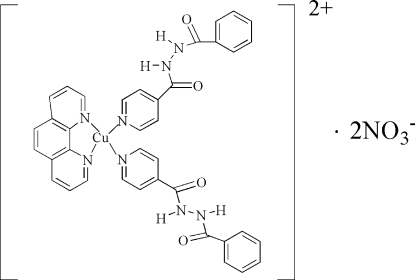


## Experimental

### 

#### Crystal data

[Cu(C_13_H_11_N_3_O_2_)_2_(C_12_H_8_N_2_)](NO_3_)_2_
*M* *_r_* = 850.26
Monoclinic, 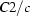

*a* = 25.126 (4) Å
*b* = 12.5304 (18) Å
*c* = 16.442 (2) Å
β = 130.827 (2)°
*V* = 3917.0 (10) Å^3^
*Z* = 4
Mo *K*α radiation
μ = 0.63 mm^−1^
*T* = 294 K
0.20 × 0.16 × 0.10 mm

#### Data collection

Bruker SMART CCD area-detector diffractometer
Absorption correction: multi-scan (*SADABS*; Sheldrick, 1996 ▶) *T*_min_ = 0.701, *T*_max_ = 1.000
10551 measured reflections
3939 independent reflections
2826 reflections with *I* > 2σ(*I*)
*R*_int_ = 0.035

#### Refinement

*R*[*F* ^2^ > 2σ(*F* ^2^)] = 0.041
*wR*(*F* ^2^) = 0.108
*S* = 1.03
3939 reflections
303 parameters
48 restraints
H atoms treated by a mixture of independent and constrained refinement
Δρ_max_ = 0.45 e Å^−3^
Δρ_min_ = −0.41 e Å^−3^

### 

Data collection: *SMART* (Bruker, 1998 ▶); cell refinement: *SAINT* (Bruker, 1998 ▶); data reduction: *SAINT*; program(s) used to solve structure: *SHELXS97* (Sheldrick, 2008 ▶); program(s) used to refine structure: *SHELXL97* (Sheldrick, 2008 ▶); molecular graphics: *SHELXTL* (Sheldrick, 2008 ▶); software used to prepare material for publication: *SHELXTL*.

## Supplementary Material

Crystal structure: contains datablocks I, global. DOI: 10.1107/S1600536810038985/vm2045sup1.cif

Structure factors: contains datablocks I. DOI: 10.1107/S1600536810038985/vm2045Isup2.hkl

Additional supplementary materials:  crystallographic information; 3D view; checkCIF report

## Figures and Tables

**Table 1:** Hydrogen-bond geometry (Å, °)

*D*—H⋯*A*	*D*—H	H⋯*A*	*D*⋯*A*	*D*—H⋯*A*
N3—H3*A*⋯O4^i^	0.81 (4)	2.15 (4)	2.873 (8)	149 (3)
N3—H3*A*⋯O4′^i^	0.81 (4)	2.43 (4)	3.20 (2)	161 (3)
N4—H4*A*⋯O3^ii^	0.83 (3)	1.99 (3)	2.814 (9)	171 (4)
N4—H4*A*⋯O3′^ii^	0.83 (3)	2.30 (4)	2.945 (18)	135 (3)
N4—H4*A*⋯O4′^ii^	0.83 (3)	2.43 (4)	3.25 (2)	172 (3)

Symmetry codes: (i) 
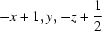
; (ii) 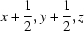.

**Fig. 1.**
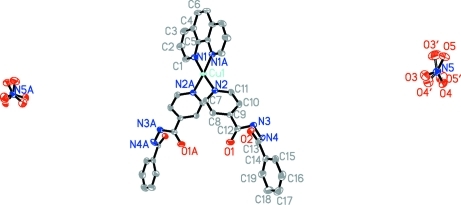
A view of the molecular structure of (I) with the atom-numbering scheme and 30% displacement ellipsoids (H atoms are omitted for clarity).

**Fig. 2.**
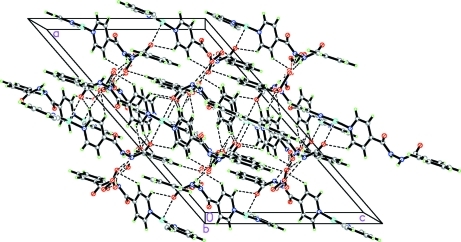
A packing diagram of the title compound. Hydrogen bonds are shown as dotted lines.

## supplementary crystallographic information

### Comment

The chemistry of hydrazine derivatives has been investigated intensively in the last decade owing to their coordinative and pharmacological activity as well as their use in analytical chemistry as metal-extracting agents (Hursthouse *et al.*, 1979; Gallego *et al.*, 1979; Haran *et al.*, 1980). As part of a continuing study (Bian *et al.*, 2005; Yu *et al.*, 2006), we have synthesized the title Cu^II^ complex, (I), and present its structure here. The molecular structure of the title compound is shown in Fig. 1. The Cu^II^ atom lying on an inversion center is coordinated by two N atoms from one phen and two pyridine N atoms of two ligands with Cu—N mean distance of 2.013 (2) Å. Therefore, the local coordination geometry of copper center is square-planar with N4 donor set. The mean deviation from the best plane through these N atoms is 0.072 (2) Å. In addition, a weak interaction exists between every Cu^II^ atom and two adjacent oxygen atoms (O2) (Cu—O2 = 2.462 (2) Å) of the ligands in the packing diagram (Fig. 2). So, four N atoms and two O atoms form an octahedral environment around the Cu^II^ atom. Four N atoms and the Cu^II^ atom form the equatorial plane, the axial position is occupied by two O2 atoms. In the compound, oxygen atoms of nitrate exhibit disorder. Three oxygen atoms are split between two sites with occupancies of 50% (O3 to O5 and O3' to O5'). The crystal packing of (I) (Fig. 2) involves N—H···O hydrogen bonds (Table 1). The nitrate O3 and O3' atoms accept intermolecular hydrogen bonds from the N4 atom of hydrazine, while O4 accepts an intermolecular hydrogen bond from the N3 atom of hydrazine and O4' interacts with both N3 and N4. These interactions and the weak interactions of Cu^II^ atoms and adjacent oxygen atoms result in a three-dimensional network of hydrogen bonds.

### Experimental

The synthesis of the ligand has been reported by Bian *et al.* (2005). *N*'-Benzoylpyridine-4-hydrazide (0.5 mmol) and Cu(NO_3_)_2_.3H_2_O (0.5 mmol) were added to the mixed solution of methanol (20 ml) and DMF (2.5 ml), meanwhile, phen (0.5 mmol) was added to the above solution. The mixture was heated and refluxed for 1.5 h, and then filtered. The filtrate was kept at room temperature for two weeks and blue crystals were obtained.

### Refinement

The oxygen atoms of the nitrate group exhibit disorder. Two sets of oxygen atoms, O3 to O5 and O3' to O5', with site-occupation factors 0.5 were refined with restraints on distances and displacement parameters. H atoms on C atoms were positoned geometrically and refined using a riding model with C—H = 0.93 Å and U_iso_(H) = 1.2U_eq_(C). H atoms on N atoms were located in a difference Fourier map.

### Crystal data

**Table:**

[Cu(C_13_H_11_N_3_O_2_)_2_(C_12_H_8_N_2_)](NO_3_)_2_	*F*(000) = 1748
*M**_r_* = 850.26	*D*_x_ = 1.442 Mg m^−^^3^
Monoclinic, *C*2/*c*	Mo *K*α radiation, λ = 0.71073 Å
Hall symbol: -C 2yc	Cell parameters from 3397 reflections
*a* = 25.126 (4) Å	θ = 2.5–26.0°
*b* = 12.5304 (18) Å	µ = 0.63 mm^−^^1^
*c* = 16.442 (2) Å	*T* = 294 K
β = 130.827 (2)°	Block, blue
*V* = 3917.0 (10) Å^3^	0.20 × 0.16 × 0.10 mm
*Z* = 4	

### Data collection

**Table:**

Bruker SMART CCD area-detector diffractometer	3939 independent reflections
Radiation source: fine-focus sealed tube	2826 reflections with *I* > 2σ(*I*)
graphite	*R*_int_ = 0.035
φ and ω scans	θ_max_ = 26.2°, θ_min_ = 2.0°
Absorption correction: multi-scan (*SADABS*; Sheldrick, 1996)	*h* = −31→26
*T*_min_ = 0.701, *T*_max_ = 1.000	*k* = −14→15
10551 measured reflections	*l* = −20→20

### Refinement

**Table:**

Refinement on *F*^2^	Primary atom site location: structure-invariant direct methods
Least-squares matrix: full	Secondary atom site location: difference Fourier map
*R*[*F*^2^ > 2σ(*F*^2^)] = 0.041	Hydrogen site location: inferred from neighbouring sites
*wR*(*F*^2^) = 0.108	H atoms treated by a mixture of independent and constrained refinement
*S* = 1.03	*w* = 1/[σ^2^(*F*_o_^2^) + (0.0397*P*)^2^ + 5.1547*P*] where *P* = (*F*_o_^2^ + 2*F*_c_^2^)/3
3939 reflections	(Δ/σ)_max_ = 0.001
303 parameters	Δρ_max_ = 0.45 e Å^−^^3^
48 restraints	Δρ_min_ = −0.41 e Å^−^^3^

### Special details

**Table:**

Geometry. All e.s.d.'s (except the e.s.d. in the dihedral angle between two l.s. planes) are estimated using the full covariance matrix. The cell e.s.d.'s are taken into account individually in the estimation of e.s.d.'s in distances, angles and torsion angles; correlations between e.s.d.'s in cell parameters are only used when they are defined by crystal symmetry. An approximate (isotropic) treatment of cell e.s.d.'s is used for estimating e.s.d.'s involving l.s. planes.
Refinement. Refinement of *F*^2^ against ALL reflections. The weighted *R*-factor *wR* and goodness of fit *S* are based on *F*^2^, conventional *R*-factors *R* are based on *F*, with *F* set to zero for negative *F*^2^. The threshold expression of *F*^2^ > σ(*F*^2^) is used only for calculating *R*-factors(gt) *etc*. and is not relevant to the choice of reflections for refinement. *R*-factors based on *F*^2^ are statistically about twice as large as those based on *F*, and *R*- factors based on ALL data will be even larger.

### Fractional atomic coordinates and isotropic or equivalent isotropic displacement parameters (Å^2^)

**Table:**

	*x*	*y*	*z*	*U*_iso_*/*U*_eq_	Occ. (<1)
Cu1	1.0000	0.30488 (4)	0.2500	0.03672 (16)	
N1	1.02696 (12)	0.18443 (17)	0.20254 (17)	0.0388 (5)	
N2	0.96446 (12)	0.42041 (17)	0.28935 (17)	0.0348 (5)	
N3	0.84202 (14)	0.6582 (2)	0.3689 (2)	0.0453 (6)	
H3A	0.8269 (17)	0.599 (3)	0.363 (2)	0.048 (10)*	
N4	0.81824 (14)	0.7421 (2)	0.3927 (2)	0.0486 (7)	
H4A	0.7939 (16)	0.786 (2)	0.343 (2)	0.047 (9)*	
O1	0.93009 (13)	0.75915 (17)	0.4076 (2)	0.0644 (7)	
O2	0.88447 (10)	0.69721 (15)	0.56691 (15)	0.0447 (5)	
C1	1.05326 (17)	0.1867 (3)	0.1541 (2)	0.0505 (8)	
H1	1.0613	0.2526	0.1378	0.061*	
C2	1.0692 (2)	0.0950 (3)	0.1270 (3)	0.0669 (10)	
H2	1.0882	0.0998	0.0940	0.080*	
C3	1.0571 (2)	−0.0020 (3)	0.1487 (3)	0.0688 (10)	
H3	1.0675	−0.0638	0.1301	0.083*	
C4	1.02878 (17)	−0.0089 (2)	0.1992 (3)	0.0534 (8)	
C5	1.01434 (14)	0.0876 (2)	0.2241 (2)	0.0405 (7)	
C6	1.0133 (2)	−0.1064 (2)	0.2252 (3)	0.0715 (11)	
H6	1.0217	−0.1711	0.2077	0.086*	
C7	1.01034 (14)	0.4875 (2)	0.3693 (2)	0.0386 (6)	
H7	1.0580	0.4778	0.4069	0.046*	
C8	0.98990 (14)	0.5703 (2)	0.3983 (2)	0.0388 (6)	
H8	1.0232	0.6166	0.4533	0.047*	
C9	0.91927 (14)	0.5841 (2)	0.3449 (2)	0.0346 (6)	
C10	0.87170 (15)	0.5150 (2)	0.2623 (2)	0.0434 (7)	
H10	0.8238	0.5222	0.2248	0.052*	
C11	0.89591 (15)	0.4351 (2)	0.2359 (2)	0.0434 (7)	
H11	0.8635	0.3898	0.1790	0.052*	
C12	0.89816 (15)	0.6754 (2)	0.3769 (2)	0.0404 (7)	
C13	0.84659 (14)	0.7618 (2)	0.4943 (2)	0.0365 (6)	
C14	0.82644 (14)	0.8668 (2)	0.5096 (2)	0.0395 (6)	
C15	0.80475 (16)	0.8715 (3)	0.5682 (3)	0.0528 (8)	
H15	0.8022	0.8095	0.5965	0.063*	
C16	0.78694 (19)	0.9682 (4)	0.5844 (3)	0.0766 (12)	
H16	0.7709	0.9712	0.6218	0.092*	
C17	0.7928 (2)	1.0596 (4)	0.5457 (4)	0.0848 (13)	
H17	0.7815	1.1248	0.5579	0.102*	
C18	0.8153 (2)	1.0565 (3)	0.4891 (3)	0.0752 (11)	
H18	0.8193	1.1193	0.4632	0.090*	
C19	0.83197 (17)	0.9595 (3)	0.4703 (3)	0.0563 (8)	
H19	0.8469	0.9569	0.4314	0.068*	
N5	0.19121 (16)	0.3709 (2)	0.1463 (2)	0.0580 (7)	
O3	0.2494 (3)	0.3972 (8)	0.2291 (5)	0.089 (2)	0.68 (2)
O4	0.1545 (3)	0.4388 (6)	0.0770 (5)	0.0651 (19)	0.68 (2)
O5	0.1665 (5)	0.2852 (5)	0.1420 (7)	0.114 (3)	0.68 (2)
O3'	0.2005 (15)	0.2750 (7)	0.1614 (14)	0.133 (7)	0.32 (2)
O4'	0.2368 (9)	0.4324 (14)	0.2155 (13)	0.084 (5)	0.32 (2)
O5'	0.1514 (9)	0.4001 (18)	0.0525 (10)	0.118 (7)	0.32 (2)

### Atomic displacement parameters (Å^2^)

**Table:**

	*U*^11^	*U*^22^	*U*^33^	*U*^12^	*U*^13^	*U*^23^
Cu1	0.0520 (3)	0.0285 (2)	0.0455 (3)	0.000	0.0388 (3)	0.000
N1	0.0464 (14)	0.0346 (12)	0.0365 (12)	0.0023 (11)	0.0276 (12)	0.0009 (10)
N2	0.0414 (13)	0.0328 (12)	0.0377 (12)	−0.0014 (10)	0.0292 (12)	−0.0010 (10)
N3	0.0562 (17)	0.0413 (15)	0.0530 (16)	0.0076 (13)	0.0421 (15)	−0.0007 (12)
N4	0.0585 (17)	0.0520 (16)	0.0421 (15)	0.0255 (14)	0.0359 (15)	0.0108 (13)
O1	0.0787 (16)	0.0349 (12)	0.0978 (19)	−0.0026 (12)	0.0657 (16)	−0.0104 (12)
O2	0.0471 (11)	0.0433 (11)	0.0404 (11)	0.0094 (10)	0.0273 (10)	0.0083 (10)
C1	0.066 (2)	0.0460 (17)	0.0539 (18)	0.0050 (16)	0.0456 (18)	−0.0009 (15)
C2	0.091 (3)	0.058 (2)	0.079 (3)	0.008 (2)	0.067 (2)	−0.0078 (19)
C3	0.080 (3)	0.053 (2)	0.077 (2)	0.0088 (19)	0.052 (2)	−0.0165 (19)
C4	0.0536 (19)	0.0376 (16)	0.061 (2)	0.0016 (15)	0.0338 (18)	−0.0098 (15)
C5	0.0367 (16)	0.0333 (15)	0.0383 (16)	0.0012 (12)	0.0187 (14)	−0.0023 (12)
C6	0.072 (3)	0.0304 (17)	0.101 (3)	0.0016 (16)	0.052 (2)	−0.0078 (18)
C7	0.0341 (14)	0.0411 (15)	0.0372 (15)	0.0008 (12)	0.0218 (13)	−0.0031 (13)
C8	0.0388 (16)	0.0377 (15)	0.0394 (15)	−0.0028 (12)	0.0254 (14)	−0.0074 (12)
C9	0.0452 (16)	0.0307 (13)	0.0356 (14)	0.0026 (12)	0.0298 (14)	0.0013 (11)
C10	0.0362 (15)	0.0533 (18)	0.0455 (17)	0.0013 (13)	0.0288 (15)	−0.0051 (14)
C11	0.0426 (17)	0.0481 (17)	0.0448 (17)	−0.0108 (14)	0.0309 (15)	−0.0154 (14)
C12	0.0471 (17)	0.0359 (16)	0.0411 (16)	0.0083 (13)	0.0301 (15)	0.0056 (12)
C13	0.0349 (15)	0.0401 (15)	0.0408 (16)	0.0044 (12)	0.0275 (14)	0.0033 (13)
C14	0.0321 (15)	0.0456 (16)	0.0376 (15)	0.0059 (13)	0.0213 (14)	−0.0023 (13)
C15	0.0445 (18)	0.061 (2)	0.058 (2)	−0.0027 (16)	0.0360 (17)	−0.0129 (17)
C16	0.063 (2)	0.087 (3)	0.092 (3)	0.003 (2)	0.056 (2)	−0.029 (3)
C17	0.075 (3)	0.066 (3)	0.093 (3)	0.015 (2)	0.046 (3)	−0.022 (2)
C18	0.078 (3)	0.047 (2)	0.078 (3)	0.0080 (19)	0.041 (2)	−0.0009 (19)
C19	0.057 (2)	0.0515 (19)	0.056 (2)	0.0067 (16)	0.0354 (18)	0.0048 (16)
N5	0.058 (2)	0.0559 (19)	0.0602 (19)	0.0132 (16)	0.0388 (18)	0.0162 (16)
O3	0.060 (3)	0.097 (4)	0.061 (3)	−0.001 (3)	0.019 (3)	0.029 (3)
O4	0.054 (3)	0.067 (3)	0.051 (3)	0.021 (2)	0.024 (2)	0.016 (2)
O5	0.115 (5)	0.061 (3)	0.173 (6)	−0.008 (3)	0.098 (4)	0.018 (3)
O3'	0.126 (11)	0.081 (8)	0.132 (9)	0.025 (6)	0.058 (7)	0.017 (6)
O4'	0.095 (8)	0.104 (9)	0.077 (8)	−0.007 (6)	0.067 (7)	−0.020 (6)
O5'	0.097 (8)	0.127 (10)	0.088 (8)	0.014 (7)	0.042 (6)	0.027 (7)

### Geometric parameters (Å, °)

**Table:**

Cu1—N1^i^	2.009 (2)	C7—H7	0.9300
Cu1—N1	2.009 (2)	C8—C9	1.383 (4)
Cu1—N2	2.017 (2)	C8—H8	0.9300
Cu1—N2^i^	2.017 (2)	C9—C10	1.378 (4)
N1—C1	1.326 (4)	C9—C12	1.494 (4)
N1—C5	1.358 (3)	C10—C11	1.381 (4)
N2—C7	1.335 (3)	C10—H10	0.9300
N2—C11	1.339 (4)	C11—H11	0.9300
N3—C12	1.346 (4)	C13—C14	1.489 (4)
N3—N4	1.387 (3)	C14—C19	1.380 (4)
N3—H3A	0.81 (3)	C14—C15	1.384 (4)
N4—C13	1.342 (4)	C15—C16	1.377 (5)
N4—H4A	0.83 (3)	C15—H15	0.9300
O1—C12	1.212 (3)	C16—C17	1.365 (6)
O2—C13	1.225 (3)	C16—H16	0.9300
C1—C2	1.384 (4)	C17—C18	1.368 (6)
C1—H1	0.9300	C17—H17	0.9300
C2—C3	1.355 (5)	C18—C19	1.384 (5)
C2—H2	0.9300	C18—H18	0.9300
C3—C4	1.407 (5)	C19—H19	0.9300
C3—H3	0.9300	N5—O3'	1.218 (8)
C4—C5	1.397 (4)	N5—O5	1.220 (5)
C4—C6	1.430 (5)	N5—O4'	1.223 (8)
C5—C5^i^	1.432 (6)	N5—O5'	1.223 (8)
C6—C6^i^	1.350 (7)	N5—O3	1.224 (5)
C6—H6	0.9300	N5—O4	1.224 (4)
C7—C8	1.375 (4)		
			
N1^i^—Cu1—N1	82.61 (13)	C8—C9—C12	118.4 (2)
N1^i^—Cu1—N2	94.72 (9)	C9—C10—C11	119.3 (3)
N1—Cu1—N2	175.04 (9)	C9—C10—H10	120.4
N1^i^—Cu1—N2^i^	175.03 (9)	C11—C10—H10	120.4
N1—Cu1—N2^i^	94.71 (9)	N2—C11—C10	122.3 (3)
N2—Cu1—N2^i^	88.24 (12)	N2—C11—H11	118.8
C1—N1—C5	117.9 (2)	C10—C11—H11	118.8
C1—N1—Cu1	130.1 (2)	O1—C12—N3	123.0 (3)
C5—N1—Cu1	111.99 (18)	O1—C12—C9	121.3 (3)
C7—N2—C11	118.2 (2)	N3—C12—C9	115.7 (2)
C7—N2—Cu1	119.31 (18)	O2—C13—N4	122.3 (3)
C11—N2—Cu1	122.49 (19)	O2—C13—C14	123.5 (2)
C12—N3—N4	117.9 (3)	N4—C13—C14	114.2 (2)
C12—N3—H3A	123 (2)	C19—C14—C15	119.7 (3)
N4—N3—H3A	118 (2)	C19—C14—C13	121.1 (3)
C13—N4—N3	121.0 (3)	C15—C14—C13	119.2 (3)
C13—N4—H4A	124 (2)	C16—C15—C14	119.9 (4)
N3—N4—H4A	114 (2)	C16—C15—H15	120.0
N1—C1—C2	122.6 (3)	C14—C15—H15	120.0
N1—C1—H1	118.7	C17—C16—C15	119.9 (4)
C2—C1—H1	118.7	C17—C16—H16	120.0
C3—C2—C1	119.8 (3)	C15—C16—H16	120.0
C3—C2—H2	120.1	C16—C17—C18	120.8 (4)
C1—C2—H2	120.1	C16—C17—H17	119.6
C2—C3—C4	119.9 (3)	C18—C17—H17	119.6
C2—C3—H3	120.1	C17—C18—C19	119.7 (4)
C4—C3—H3	120.1	C17—C18—H18	120.1
C5—C4—C3	116.6 (3)	C19—C18—H18	120.1
C5—C4—C6	118.6 (3)	C14—C19—C18	119.9 (3)
C3—C4—C6	124.8 (3)	C14—C19—H19	120.1
N1—C5—C4	123.2 (3)	C18—C19—H19	120.1
N1—C5—C5^i^	116.70 (15)	O3'—N5—O4'	119.6 (8)
C4—C5—C5^i^	120.13 (19)	O5—N5—O4'	137.8 (10)
C6^i^—C6—C4	121.26 (19)	O3'—N5—O5'	116.2 (10)
C6^i^—C6—H6	119.4	O5—N5—O5'	103.0 (10)
C4—C6—H6	119.4	O4'—N5—O5'	118.7 (8)
N2—C7—C8	122.6 (3)	O3'—N5—O3	96.3 (10)
N2—C7—H7	118.7	O5—N5—O3	119.7 (5)
C8—C7—H7	118.7	O5'—N5—O3	136.0 (10)
C7—C8—C9	119.3 (3)	O3'—N5—O4	143.3 (8)
C7—C8—H8	120.4	O5—N5—O4	120.8 (5)
C9—C8—H8	120.4	O4'—N5—O4	96.1 (10)
C10—C9—C8	118.3 (2)	O3—N5—O4	118.3 (5)
C10—C9—C12	123.3 (3)		
			
N1^i^—Cu1—N1—C1	179.2 (3)	C7—C8—C9—C10	−1.5 (4)
N2^i^—Cu1—N1—C1	−5.0 (3)	C7—C8—C9—C12	−179.3 (2)
N1^i^—Cu1—N1—C5	0.20 (14)	C8—C9—C10—C11	0.0 (4)
N2^i^—Cu1—N1—C5	176.00 (19)	C12—C9—C10—C11	177.7 (3)
N1^i^—Cu1—N2—C7	113.3 (2)	C7—N2—C11—C10	−1.5 (4)
N2^i^—Cu1—N2—C7	−62.73 (18)	Cu1—N2—C11—C10	−179.3 (2)
N1^i^—Cu1—N2—C11	−69.0 (2)	C9—C10—C11—N2	1.5 (4)
N2^i^—Cu1—N2—C11	115.0 (2)	N4—N3—C12—O1	2.8 (4)
C12—N3—N4—C13	−85.3 (4)	N4—N3—C12—C9	−177.0 (2)
C5—N1—C1—C2	−1.2 (5)	C10—C9—C12—O1	−144.4 (3)
Cu1—N1—C1—C2	179.8 (3)	C8—C9—C12—O1	33.4 (4)
N1—C1—C2—C3	0.9 (6)	C10—C9—C12—N3	35.5 (4)
C1—C2—C3—C4	−0.4 (6)	C8—C9—C12—N3	−146.8 (3)
C2—C3—C4—C5	0.3 (5)	N3—N4—C13—O2	−13.9 (4)
C2—C3—C4—C6	179.5 (4)	N3—N4—C13—C14	167.6 (3)
C1—N1—C5—C4	1.1 (4)	O2—C13—C14—C19	131.8 (3)
Cu1—N1—C5—C4	−179.8 (2)	N4—C13—C14—C19	−49.8 (4)
C1—N1—C5—C5^i^	−179.7 (3)	O2—C13—C14—C15	−45.4 (4)
Cu1—N1—C5—C5^i^	−0.6 (4)	N4—C13—C14—C15	133.1 (3)
C3—C4—C5—N1	−0.6 (5)	C19—C14—C15—C16	1.8 (5)
C6—C4—C5—N1	−179.9 (3)	C13—C14—C15—C16	179.0 (3)
C3—C4—C5—C5^i^	−179.8 (3)	C14—C15—C16—C17	−2.1 (5)
C6—C4—C5—C5^i^	0.9 (5)	C15—C16—C17—C18	1.0 (6)
C5—C4—C6—C6^i^	−1.4 (7)	C16—C17—C18—C19	0.2 (6)
C3—C4—C6—C6^i^	179.4 (5)	C15—C14—C19—C18	−0.5 (5)
C11—N2—C7—C8	−0.1 (4)	C13—C14—C19—C18	−177.7 (3)
Cu1—N2—C7—C8	177.8 (2)	C17—C18—C19—C14	−0.5 (5)
N2—C7—C8—C9	1.6 (4)		

Symmetry codes: (i) −*x*+2, *y*, −*z*+1/2.

### Hydrogen-bond geometry (Å, °)

**Table:**

*D*—H···*A*	*D*—H	H···*A*	*D*···*A*	*D*—H···*A*
N3—H3A···O4^ii^	0.81 (4)	2.15 (4)	2.873 (8)	149 (3)
N3—H3A···O4'^ii^	0.81 (4)	2.43 (4)	3.20 (2)	161 (3)
N4—H4A···O3^iii^	0.83 (3)	1.99 (3)	2.814 (9)	171 (4)
N4—H4A···O3'^iii^	0.83 (3)	2.30 (4)	2.945 (18)	135 (3)
N4—H4A···O4'^iii^	0.83 (3)	2.43 (4)	3.25 (2)	172 (3)

Symmetry codes: (ii) −*x*+1, *y*, −*z*+1/2; (iii) *x*+1/2, *y*+1/2, *z*.
